# Automatic skin lesion area determination of basal cell carcinoma using optical coherence tomography angiography and a skeletonization approach: Preliminary results

**DOI:** 10.1002/jbio.201900131

**Published:** 2019-06-18

**Authors:** Kristen M. Meiburger, Zhe Chen, Christoph Sinz, Erich Hoover, Michael Minneman, Jason Ensher, Harald Kittler, Rainer A. Leitgeb, Wolfgang Drexler, Mengyang Liu

**Affiliations:** ^1^ Biolab, Department of Electronics and Telecommunications Politecnico di Torino Torino Italy; ^2^ Center for Medical Physics and Biomedical Engineering Medical University of Vienna Vienna Austria; ^3^ Department of Dermatology Medical University of Vienna Vienna Austria; ^4^ Insight Photonic Solutions, Inc. Lafayette CO

**Keywords:** basal cell carcinoma, computer‐aided detection, optical coherence tomography angiography, quantification, skeletonization

## Abstract

Cutaneous blood flow plays a key role in numerous physiological and pathological processes and has significant potential to be used as a biomarker to diagnose skin diseases such as basal cell carcinoma (BCC). The determination of the lesion area and vascular parameters within it, such as vessel density, is essential for diagnosis, surgical treatment and follow‐up procedures. Here, an automatic skin lesion area determination algorithm based on optical coherence tomography angiography (OCTA) images is presented for the first time. The blood vessels are segmented within the OCTA images and then skeletonized. Subsequently, the skeleton is searched over the volume and numerous quantitative vascular parameters are calculated. The vascular density is then used to segment the lesion area. The algorithm is tested on both nodular and superficial BCC, and comparing with dermatological and histological results, the proposed method provides an accurate, non‐invasive, quantitative and automatic tool for BCC lesion area determination.
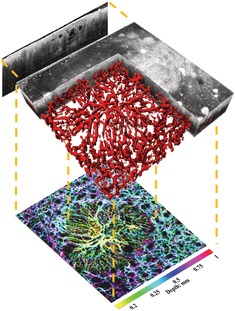

## Graphical abstract

Cutaneous blood flow is fundamental in various physiological and pathological processes and could be used as a biomarker to diagnose skin diseases, such as basal cell carcinoma (BCC). The determination of the lesion area and quantitative vascular parameters is essential for diagnosis, surgical treatment and follow‐up procedures. Here, a completely automated solution for BCC lesion area determination is presented, and quantitative vascular parameters are calculated within and outside the lesion.

## 1 INTRODUCTION

Basal cell carcinoma (BCC) is the most common type of skin cancer in the Caucasian population 1. Pathogenesis of BCC is closely linked to UV exposure and often occurs in readily visible areas of the skin. According to clinical features, BCCs are mainly classified as nodular (the most common clinical subtype), superficial (the second most common subtype) and morpheaform (accounting to 5%‐10% of the cases) 2.

To diagnose BCC, imaging has traditionally been done using a magnifying lens. More recently, dermatoscopy has been employed in routine clinical work, as it is particularly suitable for diagnosis of pigmented lesions and provides important morphological insights for a range of skin pathologies 3. Biopsies remain the gold standard for diagnosis of BCC and provide highly detailed images of the lesion. The biopsy sample is used for diagnosis and gives information about the depth of the tumor invasion. During the surgical resection, the width of the excisional area is given by dermatoscopy and the depth information is provided by biopsies 4, 5. Owing to the invasive nature of excision, along with their inability to provide longitudinal data of the same lesion, and the risk of adverse effects and scarring, biopsies are not ideal for monitoring the effect of local therapy of BCC with chemotherapeutic and immune‐modulating agents. Non‐invasive, more accurate imaging techniques are therefore highly urgent. Magnetic resonance imaging 6, computed tomography angiography 7 and ultrasound 8 have been reported to visualize skin vasculature, but they have limited resolution which is not high enough to reveal the microvasculature in the superficial layer of skin.

Optical coherence tomography (OCT) is a powerful imaging technique that enables non‐invasive, in vivo, high‐resolution, cross‐sectional imaging in biological tissues. OCT systems can achieve axial and lateral resolutions of a few micrometers 9, 10. A promising field of application for OCT is in dermatology, where it has two distinct virtues: whereas dermatoscopy provides a high‐resolution en face view of the skin surface, OCT offers cross‐sectional imaging revealing tissue morphology down to a depth of 1 to 2 millimeters. In addition, compared with the gold standard histology, non‐invasiveness of OCT allows repeated imaging sessions to monitor pathogenesis and therapy over time without the need of taking tissue samples 3. Recently, it has been proved that OCT can be used in the diagnosis of BCC 11, 12. However, there are numerous other features that can be mistaken for tumors in OCT images, such as hair follicles, cysts and benign neoplasms. Lacking the specificity needed for BCC diagnosis, morphological imaging using OCT alone is not enough. In view of this, a functional extension of OCT, named OCT angiography (OCTA), may fill in the gap by giving vasculature information of the underlying lesion and surrounding areas.

By acquiring B‐scans at the same position for multiple times, OCTA can contrast the pixels corresponding to blood vessels using the intensity and/or phase modulation exerted by moving hemoglobin in the vessels 13. Currently, OCTA is the most successful functional extension of OCT, since it can be implemented in any OCT platform and it meets an immediate clinical diagnostic need 14, 15. Knowing that in vivo visualization of cutaneous blood vessels may aid in diagnosis and treatment of dermatological disorders 16, in the past few years, OCTA has been actively applied in dermatological research for a variety of skin diseases 17. By performing an OCTA scan on skin, both tissue and vascular morphologies can be extracted in a non‐invasive manner in just a few seconds. The specific appearance of cutaneous vasculature gives clinicians a valuable reference for their initial diagnosis of skin diseases.

However, it is of fundamental importance to be able to extract not only qualitative information, but also quantitative parameters that can objectively describe the complexity of the examined blood vessels 18. Extracting quantitative information from OCTA volumes is a recent hot topic in research, and various studies exist in literature, many of which focus on retinal microvasculature 19, 20, 21, 22, preclinical animal models 23, 24, 25, and specific dermatological cases 26, 27, 28, 29, 30, 31. Among the various methods to assess the complex vasculature, skeletonization techniques have been shown to characterize and quantify vascularization in numerous clinical and preclinical applications using different imaging modalities, such as contrast‐enhanced ultrasound 32, 33, acoustic angiography 34, photoacoustics 35 and more recently also in OCTA 36, 37. Skeletonization is based on an initial segmentation of the vasculature, which is then reduced to a minimal representation, the skeleton. The skeleton can then be automatically searched, and quantitative parameters can be calculated within specific regions‐of‐interest (ROI). A disadvantage of this approach is that the ROI is typically placed manually on the volume to determine the quantitative parameters within an area that is decided by the user, generating inter‐ and intra‐operator variability of the ROI placement.

When considering non‐dermatological applications, quantitative vascular parameters have been used for studying retinal microvascular changes in uveitis and showed how skeleton‐based OCTA algorithms are robust enough to detect changes in uveitis subjects 3. The technique was, however, only semi‐automatic, and the entire imaged region was considered either healthy or diseased, precluding the need for distinguishing a lesion area within the imaged volume.

When considering dermatological applications, a correlation mapping mask was proposed for improving microcirculation imaging of human skin and the method quantitatively assessed the difference between a burn scar and normal skin of one subject using the vessel area density parameter, showing how this parameter can distinguish between the two skin conditions 38. The repeatability of the vessel density measurement in human skin using OCTA volumes acquired on healthy individuals has been presented recently 39. In their study, the authors acquired three different OCTA volumes on four locations (volar wrist, volar forearm, shoulder, and volar upper arm) and then segmented the volumes and calculated the vessel density of each skin layer (papillary dermis, reticular dermis and the whole dermis layer). Their results showed that the quantification of vessel density using OCTA volumes is repeatable in healthy individuals. It is shown in another study that human skin wound healing can be monitored over time using OCTA by extracting the vessel diameter and density and may help to ascertain wound severity and possible healing outcomes 30.

In this paper, we demonstrate an automatic skin lesion area determination algorithm based on the OCTA data of BCC. This algorithm firstly extracts multiple quantitative vascular parameters for the whole volume. Then the vascular density parameter is used to marginalize the lesion zone based on a parametric analysis.

## 2 MATERIALS AND METHODS

### 2.1 The OCT system

**Figure 1:**
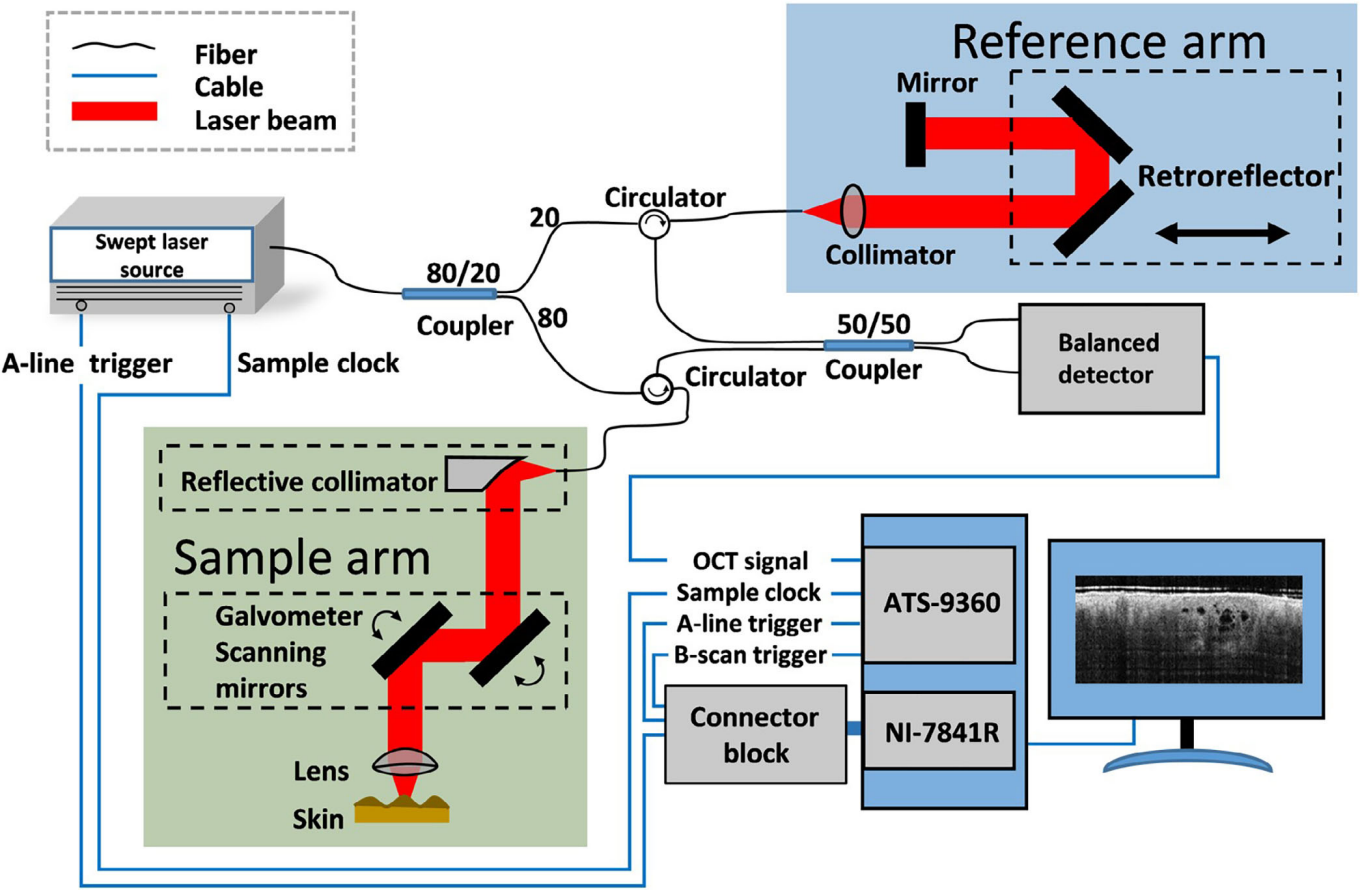
Schematic diagram of the optical coherence tomography angiography system used in the experiments

The schematic diagram of the OCT system is shown in Figure 1. The system employs a swept source with a central wavelength of 1340 nm and a bandwidth of 37 nm running at the sweep repetition rate of 200 kHz (SSOCT‐1340; Insight Photonic Solutions, Inc). The power incident upon the sample is measured to be 5.8 mW. The data are acquired by a digitizer (ATS9360, Alazar Technologies, Inc.) at 400 MS/s. The lateral resolution is 54.64 μm in air. The axial resolution is 26.86 μm in air (18.52 μm in tissue). The horizontal imaging range is 10 mm × 10 mm, and the penetration depth is around 1.2 mm. With a customized imaging unit, the system can access various parts of the patients' bodies. For details of the system please refer to previous studies 14, 40.

The post‐processing employs a complex signal–based OCTA algorithm 41, 42. Motion contrast is achieved by calculating the difference of consecutive B‐scans in the same position. Four consecutive B‐scans in the same position are used in our system and numerical stabilization algorithms are used to improve the phase stability.

### 2.2 Human subjects

Seven patients with BCC were imaged. One of them presented a nodular BCC (nBCC) on the head. The other six presented superficial BCCs (sBCCs) on the leg or the arm. The age of the patients ranged from 30 to 70 years, and both genders were recruited.

The experimental procedure was approved by the Ethics Committee of the Medical University of Vienna (EK 1246/2013). Informed consent was obtained from participating subjects prior to the experiment.

### 2.3 Lesion area determination algorithm

**Figure 2:**
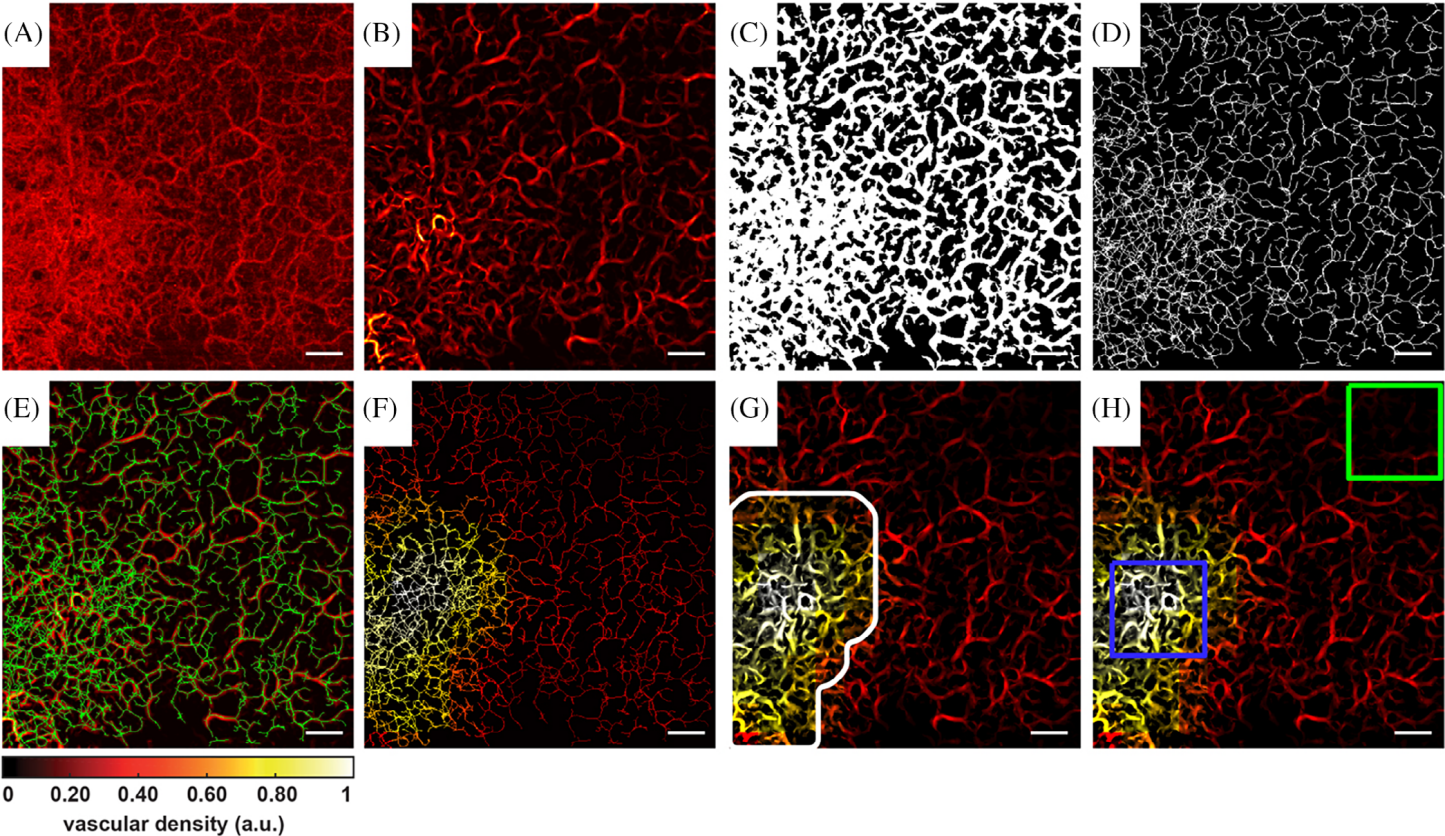
Image processing steps. All sub‐figures are given as en face view maximum intensity projection images. Scale bar: 1 mm. A, Original optical coherence tomography angiography volume. B, Preprocessed volume. C, Segmented volume. D, Skeleton volume. E, Preprocessed volume with skeleton overlaid in green. F, Skeleton volume presented in vascular density heat map. G, Preprocessed volume presented in vascular density heat map. The lesion area is encircled by the white line. H, Heat map of the preprocessed volume with the lesion area (blue square) and healthy area (green) regions‐of‐interests. Bottom right: heat map scale for normalized vascular density

Figure 2 outlines the main steps of the algorithm, which are described in further detail in the following sections. The automatic skin lesion area determination algorithm relies on the calculation of three‐dimensional (3D) quantitative vascular parameters on the skeletonized vasculature. To acquire a correct skeleton, an accurate segmentation of the vessels is required. Hence, the acquired OCTA volume was first preprocessed and then a threshold was used to obtain the segmentation. The skeleton was obtained from the automatic segmentation and subsequently, an automatic lesion area determination algorithm was applied to determine the location of the BCC lesions.

#### 2.3.1 Image preprocessing and segmentation

One OCTA volume includes 512 B‐scans, 490 A‐lines with 410 pixels per A‐line. This corresponds to a volume size of 10 mm × 9.57 mm (horizontal direction) × 1.35 mm (in depth). Most vasculature visible in the OCTA volumes is in a specific depth range, so first of all, a 0.3 mm depth window containing most of the vasculature was isolated from the acquired volume to reduce the computational time. This was done automatically by calculating the intensity of the OCTA signal in all the slices and finding the most superficial slice and the deepest slice that included the entire intensity distribution the best.

Subsequently, a 3D median filter (kernel size 3 × 3 × 3 pixels) was applied on the raw data to reduce the noise present in the volume. Then, a Frangi vesselness filter was applied to the volume since the objects of interest in the volume are blood vessels 43. The Frangi filter simultaneously reduces noise in the volume that does not correspond to blood vessels and enhances the vessels. This filter is characterized by the scale that determines the dimensions of the vessels that are recognized and enhanced in the 3D volume. It is possible to combine multiscale measurements and therefore recognize both larger and smaller vessels. In this study, we adopted a scale size ranging from 1 to 5 with a step size of 1 to enhance both capillaries and vessels with a larger diameter. The filtered maximum intensity projection (MIP) en face image can be seen in Figure 2B.

To obtain a final binary volume where white corresponds to vessels, the preprocessed slices of the volume must be correctly segmented. Each slice clearly represents either the lack of an OCTA signal (hence structures not containing moving hemoglobin) or the presence of a signal, that corresponds to structures containing flowing hemoglobin. Therefore, in order to segment the vessels, a fast adaptive thresholding technique 44 was employed slice by slice in order to reconstruct the final 3D volume. The final segmented en face MIP view can be seen in Figure 2C.

#### 2.3.2 Skeletonization

After the segmentation, a 3D skeletonization method was employed on the segmented volume to reduce the representation of the vascular network. Skeletonization techniques are designed to reduce the segmented binary volume into a minimal representation of the vascular network while still preserving its morphology. Specifically, a medial axis extraction algorithm 45, 46 was used since the morphology of interest in this study is that of the blood vessels. Hence, the final skeleton represents the medial axis of the segmented vessels. The en face view of the obtained skeleton is shown in Figure 2D. The final skeleton overlaid on the OCTA MIP en face view is displayed in Figure 2E.

#### 2.3.3 Vascular parameter extraction & lesion area determination

The obtained 3D skeletons can be used to analyze the characteristics of the vessel network thanks to a skeleton search algorithm and the calculation of quantitative vascular parameters within a specific ROI. In this work, seven quantitative parameters are considered, which are as follows:

1. Number of trees: number of independent vascular trees into which the search algorithm decomposes the skeleton volume.
2. Vascular density (VD): number of skeleton voxels in a unit volume within the ROI.
3. Number of branches (NB): number of branches found within the structure.
4. Mean radius (MR): MR of the vessels found within the ROI.
5. Two‐dimensional distance metric (DM): defined as the ratio between the actual path length of a considered vessel and the linear distance between the first and the end points of that vessel. This measures the bidimensional tortuosity of the vessel, given that a straight line would yield a DM of 1, whereas as the curvature of the vessel increases, so does the DM.
6. Inflection count metric (ICM): defined as the DM multiplied by the number of inflection points found along the vessel path. This parameter considers not only the overall curvature of the vessel but also the number of times the vessel changes direction in its path.
7. Sum of angles metric (SOAM): defined as the sum of all the angles that a curve has in space. This tortuosity parameter is helpful in the case of tightly coiled vessels, which are not well‐represented by either the DM or ICM.

As mentioned in the introduction, these quantitative vascular parameters are typically calculated within manually placed volume ROIs, usually considering a healthy area and a lesion area. Here, we instead present a completely automatic approach to determine the skin lesion area in BCC. To achieve this, the following procedure was used:

- A grid of ROIs was generated with a 30% overlap in the horizontal and vertical dimension between juxtaposed ROIs. Each of the ROIs corresponded to a volume size of 2.5 mm × 2.5 mm × 0.3 mm.
- The quantitative vascular parameters were calculated for each ROI.
- A skeleton and a vessel volume were obtained for each vascular parameter. As an example, Figure 2F,G show the skeleton volume and the preprocessed volume as a heat map of the VD, respectively. These volumes are no longer binary; rather, the previously considered binary object (ie, the skeleton or the vessel segmentation) assumes the value of the calculated vascular parameter. The overlap of ROIs was introduced to allow a smoother transition from different values of the quantitative parameters; in fact, in the areas that were overlapped, the average value of the quantitative vascular parameter is reported as the final value. The general flowchart of this approach is detailed in Figure S1 in the supplementary material.

For BCC lesions, it was found that the VD parameter can be used to discriminate the lesion area, which can be determined by setting a threshold on the heat map of the VD.

First of all, however, it is necessary to consider the case that a lesion may not always be present within the OCTA volume. In the case of no lesion present within the acquired volume, it can be assumed that the underlying vasculature presents a similar architecture and distribution within the entire volume. Therefore, to discriminate the case of no BCC lesion present, the SD of all of the VD values calculated within the 3D VD skeleton was calculated. In the case of no lesion, this value is expected to be small since there is an even distribution of vascular density throughout the volume, so a minimum value of 4 × 10^−5^ was used.

Once determining if a lesion is actually present, the lesion area determination algorithm then does an initial thresholding on the heat map of the VD, using a value equal to 75% of the maximum. A check is then done to determine if the BCC lesion is superficial or nodular. In the case of a sBCC, the areas that present a high vascular density correspond to the lesion area, so only one potential lesion area is found with this initial threshold. In the case of a nBCC, on the other hand, the lesion is represented by a low vascular density, so numerous potential lesion areas are found with the initial threshold. Due to the different characteristics of these two lesions (ie, sBCC or nBCC), two different processing methods were used to determine the final lesion area, once they were correctly distinguished.

- For sBCC, after comparing with the results given by biopsies along with dermatologists' judgment, the proper threshold was chosen to be equal to 75% of the maximum value in the heat map. This area was then enlarged and smoothed using a morphological operation (disk structural element, radius 20). Figure 2G indicates the lesion area over the whole scanning range in an en face view.
- For nBCC, an iterative process was followed by thresholding the VD heat map using a threshold equal to 20% higher than the non‐zero minimum value. If two or more distinct areas were found in the volume, the threshold was increased by 30% until the final lesion area was determined. This area was then enlarged and smoothed using a morphological operation (disk structural element, radius 20).

Once the lesion area was determined, the seven quantitative vascular parameters were calculated within a ROI (2.5 mm × 2.5 mm × 0.3 mm) centered on the maximum of the VD heat map. Subsequently, the healthy area in which to calculate the same quantitative vascular parameters was determined as the ROI in the volume that was found to be the furthest away from the lesion area, which is demonstrated in Figure 2H. The flowchart of the lesion area determination procedure is detailed in Figure S2 in the supplementary material.

## 3 RESULTS AND DISCUSSION

### 3.1 Validation of the automatic segmentation algorithm

In order to validate the proposed algorithm, we compared the results from the automatic algorithm with those obtained using a semi‐automatic segmentation method provided by a commercial software (Thermo Scientific Amira, version 6.4.0). The semi‐automatic method can select the pixels corresponding to a branch of vessel that is associated with similar intensities 47. By manually choosing the branches one by one, the whole blood vessel network can be segmented. After the semi‐automatic segmentation, the same procedures as described in Sections 2.3.2 and 2.3.3 are applied for skeletonization, vascular parameter extraction, and finally lesion area determination.

We then calculated the vascular parameters within the determined lesion and healthy areas for both the completely automatic method and the semi‐automatic method. The parameters generated by these two methods were then compared using a paired Student's t‐test. The centroid of the lesion area obtained by both methods was also compared to verify the localization of the determined lesion areas. The intra‐operator variability of the semi‐automatic method was assessed by letting the same operator perform the same task at two different time points 1 week apart (T1 and T2, where T2 = T1 + 7 days).

Owing to the complexity of the vessel network, we chose to validate the segmentation against a semi‐automatic approach, considering that a manual segmentation would be very time‐consuming and be prone to errors due to the presence of microvasculature and noise. Furthermore, the main focus of this work is the development of a lesion area determination algorithm and not a vessel segmentation algorithm, hence we did not perform a pixel‐based validation of the vessel segmentation with the semi‐automatic algorithm but preferred to focus on the calculation of the quantitative vascular parameters.

**Figure 3:**
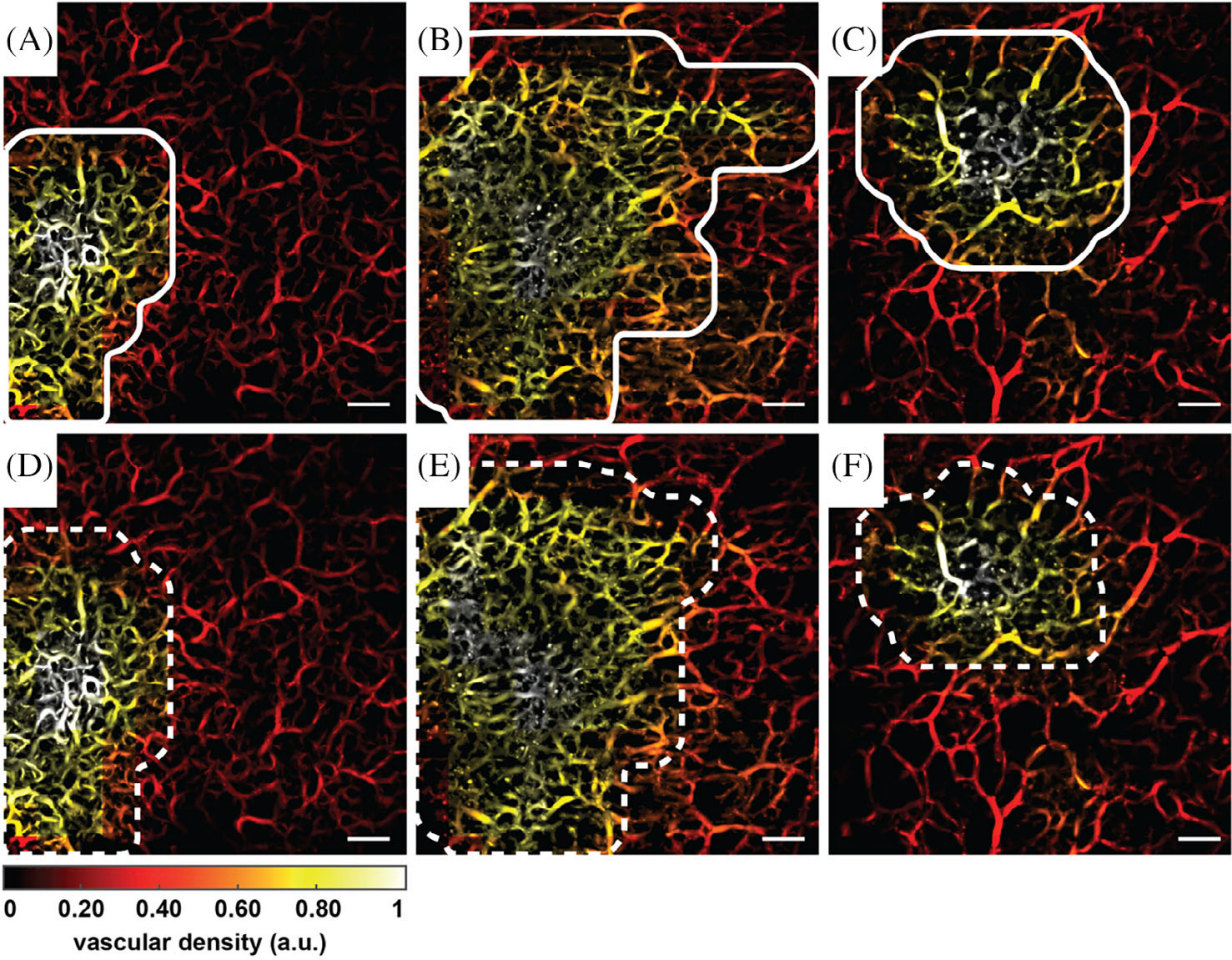
Completely automatic and semi‐automatic lesion area determination. Scale bar: 1 mm. Each image is shown as an en face view of the vascular density (VD) generated heat map. The upper row (panels A, B, C) shows the results generated by the completely automatic algorithm with the lesion area encircled by a solid white line while the lower row (panels D, E, F) shows the results by the semi‐automatic method with the lesion area encircled by the dashed white line. Bottom left: heat map scale for normalized VD

Figure 3 demonstrates how the automatic algorithm determines the lesion area very closely to the semi‐automatically determined area.

The averaged quantitative vascular parameters calculated in the automatically determined lesion and healthy areas for all seven cases are shown in the first and the second rows of Table 1, respectively. The third and the fourth rows are the quantitative vascular parameters calculated using the semi‐automatic method for T1. The last two rows are the same as the third and the fourth rows but for T2. Finally, the average distance between the centroids of the lesion areas calculated by the automatic and semi‐automatic methods was found to be 0.59 ± 0.42 mm.

**Table 1:** Quantitative vascular parameter results

	NT^a^^,^ b	NB^a^^,^ b ^,^ c ^,^ e ^,^ f	VD^a^^,^ b ^,^ c ^,^ d ^,^ e ^,^ f	MR^a^^,^ b ^,^ c ^,^ d ^,^ e [mm]	DM	ICM	SOAM^a^^,^ c ^,^ d
Auto lesion area	1.29 ± 0.49	148.86 ± 31.47	0.002 ± 0.000	0.115 ± 0.007	2.56 ± 0.32	70.31 ± 19.25	0.29 ± 0.04
Auto healthy area	3.00 ± 1.29	91.71 ± 40.01	0.001 ± 0.001	0.145 ± 0.017	2.27 ± 0.20	55.05 ± 19.50	0.21 ± 0.08
Semi‐auto lesion area T1	1.57 ± 0.79	193.43 ± 69.00	0.003 ± 0.001	0.099 ± 0.006	2.46 ± 0.20	64.58 ± 13.82	0.32 ± 0.05
Semi‐auto healthy area T1	2.14 ± 1.22	108.29 ± 21.26	0.001 ± 0.000	0.131 ± 0.015	2.48 ± 0.16	63.74 ± 16.52	0.26 ± 0.04
Semi‐auto lesion area T2	1.00 ± 0.00	239.29 ± 59.82	0.004 ± 0.001	0.093 ± 0.010	2.44 ± 0.26	63.55 ± 13.93	0.31 ± 0.06
Semi‐auto healthy area T2	2.00 ± 1.00	145.27 ± 33.61	0.002 ± 0.001	0.116 ± 0.009	2.38 ± 0.14	58.69 ± 12.74	0.30 ± 0.07

a Statistically significant difference between automatic lesion and healthy area.

b Statistically significant difference between semi‐automatic lesion and healthy area at time point 2 (T2, with T2 = T1 + 7 days).

c Statistically significant difference between semi‐automatic lesion and healthy area at time point 1 (T1).

d Statistically significant difference between automatic and semi‐automatic lesion area (T1).

Statistically significant difference between automatic and semi‐automatic healthy area (T1).

e Statistically significant difference between semi‐automatic lesion area T1 and T2.

f Statistically significant difference between semi‐automatic healthy area T1 and T2.

### 3.2 Validation of the lesion area determination algorithm

**Figure 4:**
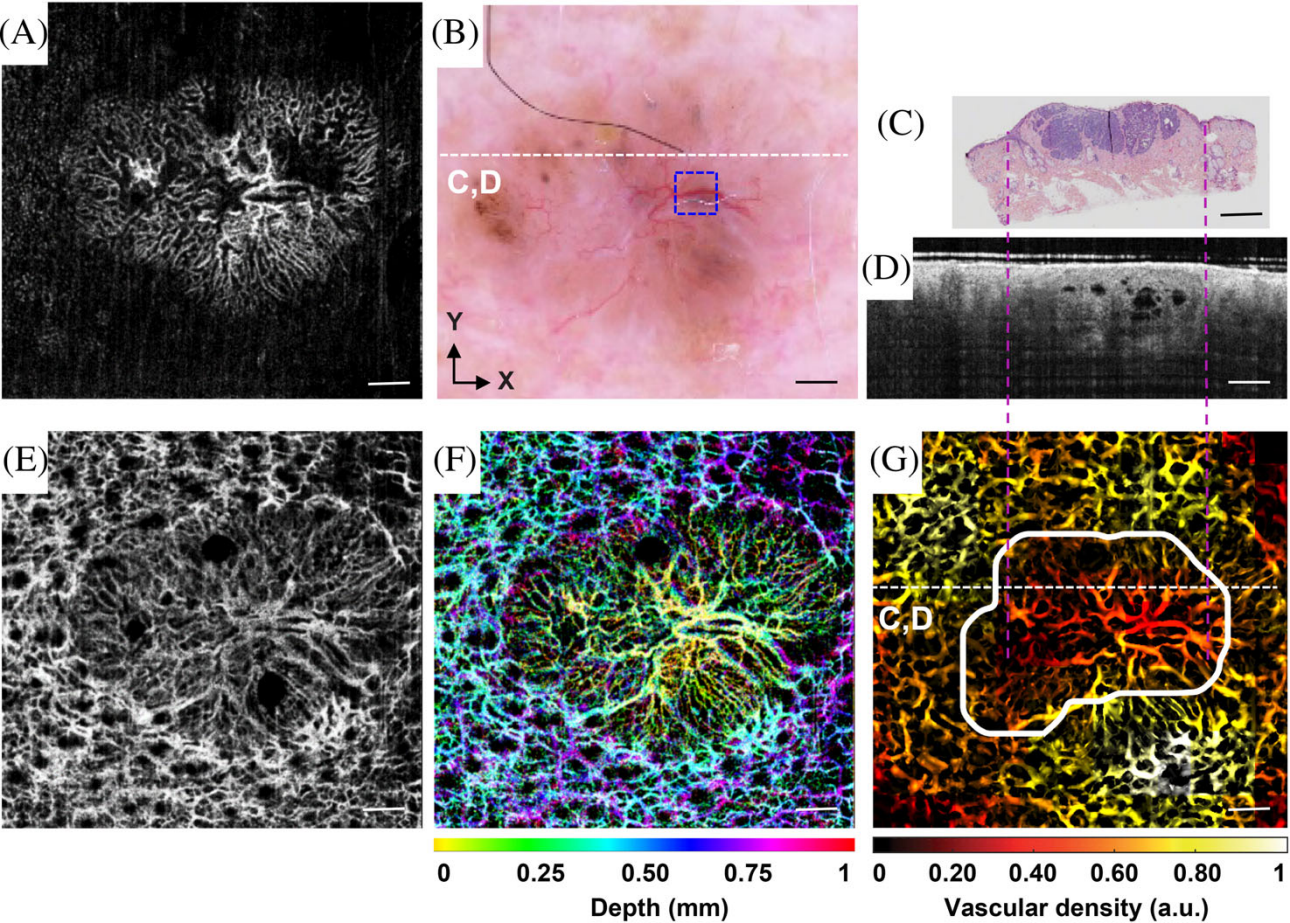
Validation of the automatic method using the nodular basal cell carcinoma case. Scale bar: 1 mm. A, optical coherence tomography angiography (OCTA) maximum intensity projection (MIP) image for the depth range between 0.1 and 0.5 mm below skin surface. B, Dermatoscopy image of the lesion. C, One biopsy image. Dark purple area in the center indicates the lesion. D, B scan given by OCT. C, OCTA MIP image for the depth range between 0.5 and 1 mm below skin surface. F, OCTA MIP image with depth color coding from 0.1 to 1 mm. G, vascular density heat map with the automatically determined lesion area encircled by the white line. The dotted white lines in (B) and (G) indicate the position of the cross section shown in (C) and (D)

To demonstrate the accuracy of the automatic lesion area determination algorithm, a validation with histological results using the nBCC case was performed. For this case, firstly a dermatoscopy examination was given. Then the area of interest was scanned by OCTA. Finally, three biopsies were taken. The first biopsy was taken at the position where the thickest blood vessel is found in the blue dashed square in Figure 4B in the x axis direction. The other two biopsies were taken with a ± 1 mm shift in the y axis relative to the position of the first biopsy.

From the dermatoscopy image shown in Figure 4B, we can only see a few superficial blood vessels, which are not enough to accurately set the margin of the lesion. The white dashed line in Figure 4B marks the approximate position where one biopsy was taken. Figure 4C shows the histological findings in a cross‐sectional image where the lesion is indicated by the color of dark purple. The corresponding OCT B‐scan is shown in Figure 4D. Figure 4G indicates the automatically determined lesion area by the white line in an en face view. The purple dashed lines across Figure 4C,D,G mark the approximate span of the lesion in the x axis direction. As can be seen, the OCT B‐scan and the automatically determined lesion area match well with the histological findings. We can also notice from Figure 4D that the upper boundary of the lesion extends to the epidermis and that the dermal‐epidermal junction fades away along this upper boundary.

Figure 4A shows the OCTA resolved vasculature in the first half millimeter in skin. Numerous micro‐vessels are seen in this image. Figure 4E shows the vasculature between 0.5 mm – 1 mm in skin. In this layer, thicker vessels which feed the microcirculation above are visualized. Figure 4F gives a depth color coding to the vessels over 1 mm. Comparing Figure 4A,E,F we can see that capillary loops in the healthy area start at a depth of about 0.3 mm while those in the lesion zone start at shallower depths. This finding agrees with the observation in Figure 4D.

**Figure 5:**
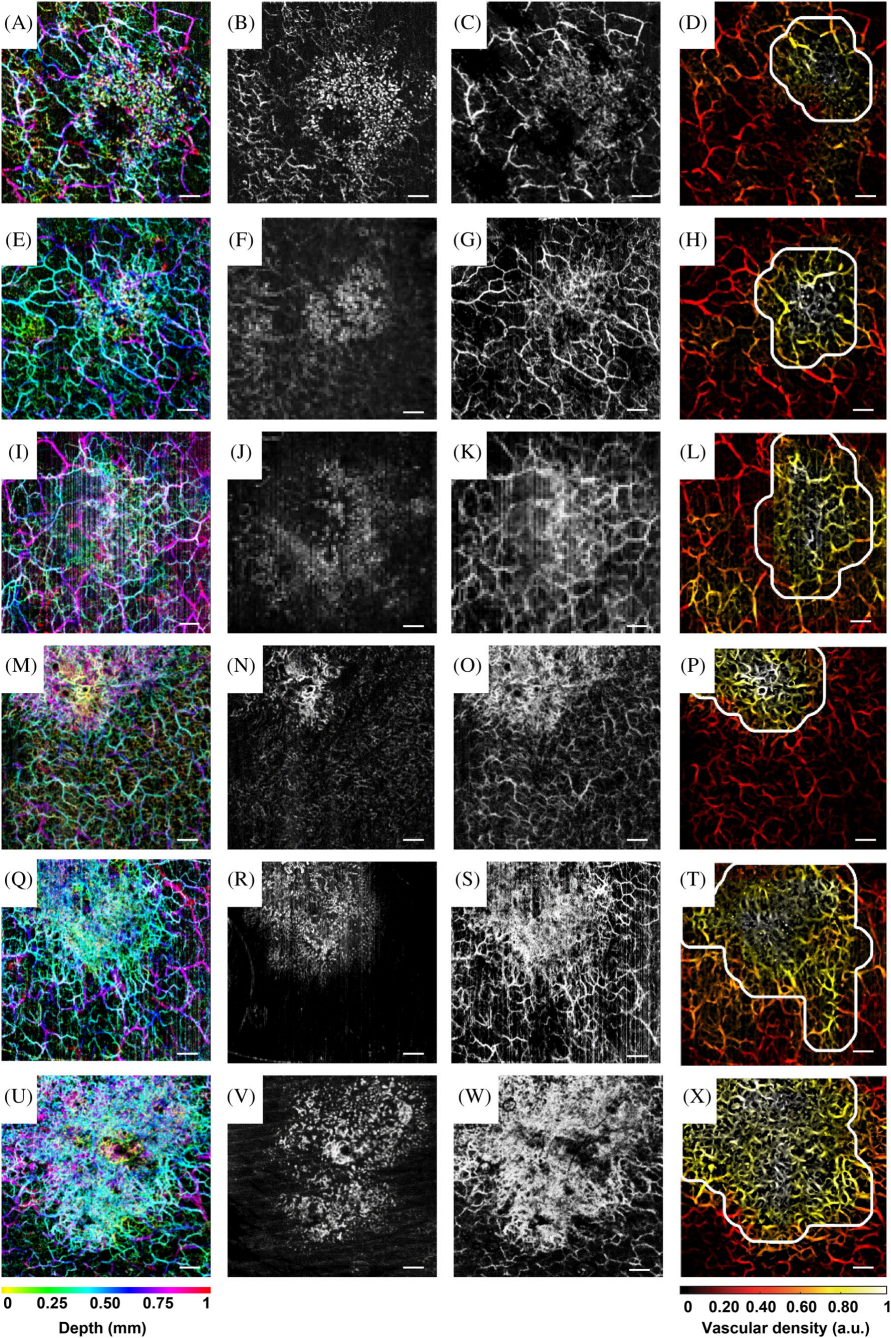
Six patients' results of superficial basal cell carcinoma. One row for one patient. Scale bar: 1 mm. (A), (E), (I), (M), (Q), (U): En face view of depth‐color‐coded images given by optical coherence tomography angiography, depth range from 0.1 to 1 mm under the skin surface. (B), (F), (J), (N), (R), (V): En face maximum intensity projection (MIP) images for the depth range from 0.1 to 0.5 mm. (C), (G), (K), (O), (S), (W): En face MIP images for depth range from 0.5 to 0.1 mm. (D), (H), (L), (P), (T), (X): vascular density heat maps for all six cases with the automatically determined lesion area encircled by the white line

The robustness of the automatic algorithm is demonstrated using the remaining six sBCC cases. The results are presented in Figure 5 with each row representing one case. The leftmost column of Figure 5 shows the depth‐color‐coded vasculature over the depth range between 0.1 and 1 mm for all six cases. The two middle columns show the MIP en face view of the vessels in depth ranges of 0.2‐0.5 mm and 0.5‐1 mm. Finally, the rightmost column displays the VD heat map with the automatically determined lesion areas encircled by the while line.

The ability to evaluate vessel network morphology and complexity in a non‐invasive and quantitative manner can provide crucial information for the diagnosis and treatment of dermatological disorders. For BCC, this study demonstrates that the developed automatic algorithm gives comparable results with those yielded by the semi‐automatic method but using much less time. Since the steps after the segmentation for determining the final lesion area are the same for both the automatic and the semi‐automatic methods, the main time difference is due to the segmentation method. In particular, the developed automatic algorithm takes less than 1 minute to segment the entire volume, whereas the semi‐automatic commercial software required up to 1 hour to provide an acceptable segmentation of the entire vessel network of one volume.

Moreover, for the NB, VD and MR parameters, statistically significant differences between the healthy and lesion areas are confirmed not only by the automatic method, but also by the semi‐automatic method at two time points. Admittedly, a statistically significant difference is noticed between the automatically and the semi‐automatically calculated VD, MR and SOAM in the lesion area, but when the healthy areas are considered, this difference is not noticed. At the same time, however, we can see how the semi‐automatic segmentation should not necessarily be considered the ground truth, as it presents a high intra‐operator variability. In fact, a statistically significant difference was found when considering the lesion area in two different time points for the NB, VD, and MR parameters; when considering the healthy area, a significant difference was found for the NB and VD parameters.

It can be appreciated how the VD and MR parameters often showed a statistically significant difference in several comparisons (ie, automatic vs semi‐automatic, or semi‐automatic in two time points). This can be explained by the fact that the program Amira allows a semi‐automatic segmentation by adjusting a threshold on the considered vessel or branch. Therefore, a slight increase or decrease of this threshold will produce a subsequent under‐segmentation (ie, vessels are thinner and at times disconnected) or over‐segmentation (ie, vessels are thicker, and more noise is present), which directly affects these two vascular parameters.

Moreover, it is important to underline that the considered vascular parameters should always be evaluated in comparison with values calculated in the same manner and in the same considered volume area, as a nominal value may change if considering a larger or smaller ROI. Moreover, as our results show, a different vessel segmentation can influence the nominal values of the quantitative vascular parameters but influences in a much smaller degree the comparison between the nominal values in the healthy and lesion areas.

Although the statistical analysis reported here shows very promising results, some limitations still exist. Firstly, more data needs to be acquired and analyzed in order to have a stronger statistical significance. Secondly, the proposed approach needs to be validated with new test datasets that were not used for the development of the method. This is specifically important for the validation of the thresholds used in the algorithm, in particular considering the threshold used for determining the presence of an actual lesion. Thirdly, the technique does not address potential errors that may be caused by the automatic segmentation and skeletonization, such as spurious branches and disconnected vessels or the presence of artifacts due to shadow graphic projection from superficial large vessels on deeper layers, also known as the tail artifact. Future work includes increasing the robustness of the developed approach by implementing advanced algorithms to reduce shadowing artifacts 48, and to remove spurious branches and connect disconnected vessels 49, 50. The accuracy of the border detection needs to be compared with other in vivo examination techniques such as confocal laser scanning microscopy and with histopathology in a prospective study. Finally, as the OCTA database size of BCC lesions and other skin diseases increases, we plan to implement machine learning techniques for the automatic classification of the lesion area and the lesion type.

## 4 CONCLUSION

In conclusion, this work presents a novel automatic method for skin BCC lesion area determination using 3D skeletonized OCTA images that can aid the diagnosis and treatment of dermatological disorders. We believe our system and algorithm will potentially have a great influence on the routine diagnosis of BCC and plan to explore the application of this technology in other skin diseases.

## Supporting information


**Figure S1.** General flowchart of the lesion area determination algorithm. ROI, region of interest; VD, vascular density.
**Figure S2.** Flowchart highlighting specific steps in the algorithm, specifically how it is determined if a lesion is present; if present, how the lesion is determined to be superficial or nodular BCC; highlighting the difference in the algorithm when considering the two different BCC lesions. VD, vascular density; BCC, basal cell carcinoma; VD_std_, SD of VD values within the skeleton; VDlim_std_, threshold value for SD of VD values within the skeleton, equal to 4 × 10^−5^ in this preliminary study.
