# Supplementary material for: Automatic skin lesion area determination of basal cell carcinoma using optical coherence tomography angiography and a skeletonization approach: Preliminary results
Source: J Biophotonics. 2019 Jun 18;12(9):e201900131. doi: 10.1002/jbio.201900131 (PMC7065618; doi:10.1002/jbio.201900131)

Supporting Information

Title: Automatic Skin Lesion Area Determination of Basal Cell Carcinoma using OCT Angiography and a Skeletonization Approach: Preliminary Results

Zhe Chen, Christoph Sinz, Erich Hoover, Michael Minneman, Jason Ensher, Harald Kittler, Rainer A. Leitgeb, Wolfgang Drexler, Mengyang Liu, and Kristen M. Meiburger

Additional supporting information may be found in the online version of this article at the publisher’s website.

**Figure S1**: General flowchart of the lesion area determination algorithm. ROI: Region of interest, VD: vascular density.


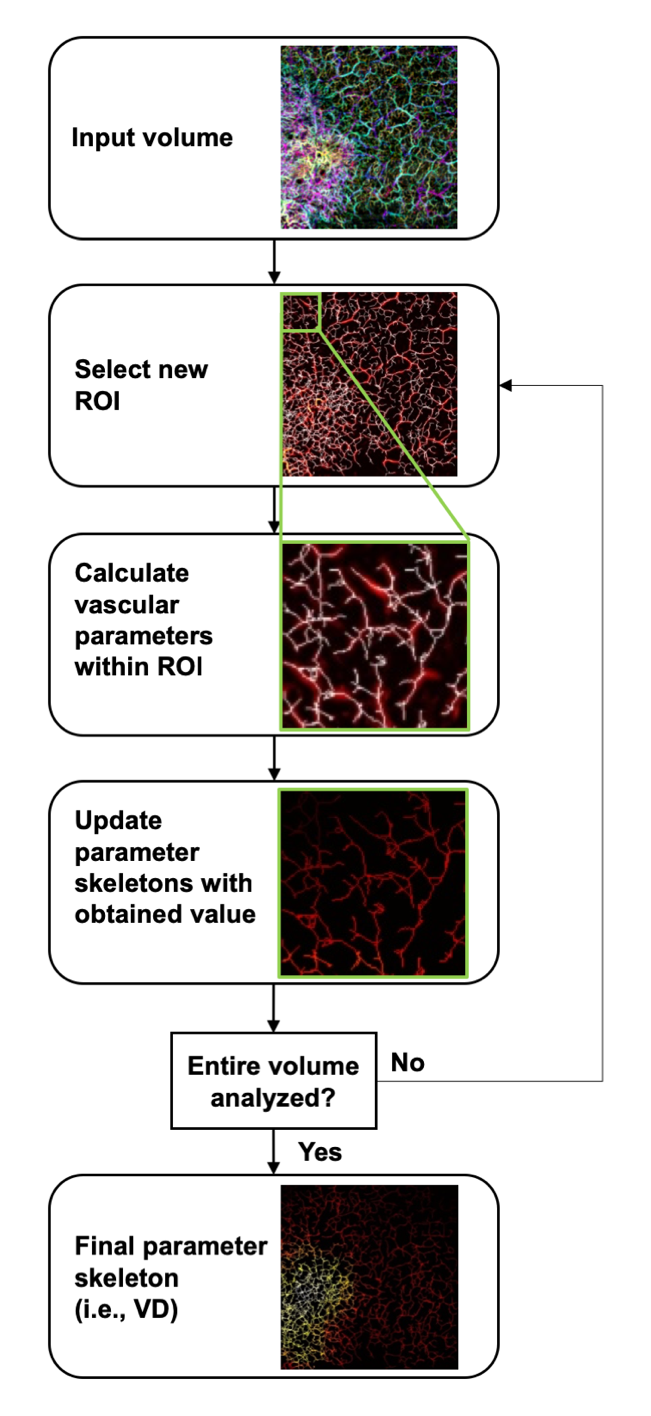


**Figure S2**: Flowchart highlighting specific steps in the algorithm, specifically how it is determined if a lesion is present; if present, how the lesion is determined to be superficial or nodular BCC; highlighting the difference in the algorithm when considering the two different BCC lesions. VD: vascular density, BCC: basal cell carcinoma, VD_std_: standard deviation of VD values within the skeleton, VDlim_std_: threshold value for standard deviation of VD values within the skeleton, equal to 4x10^-5^ in this preliminary study.


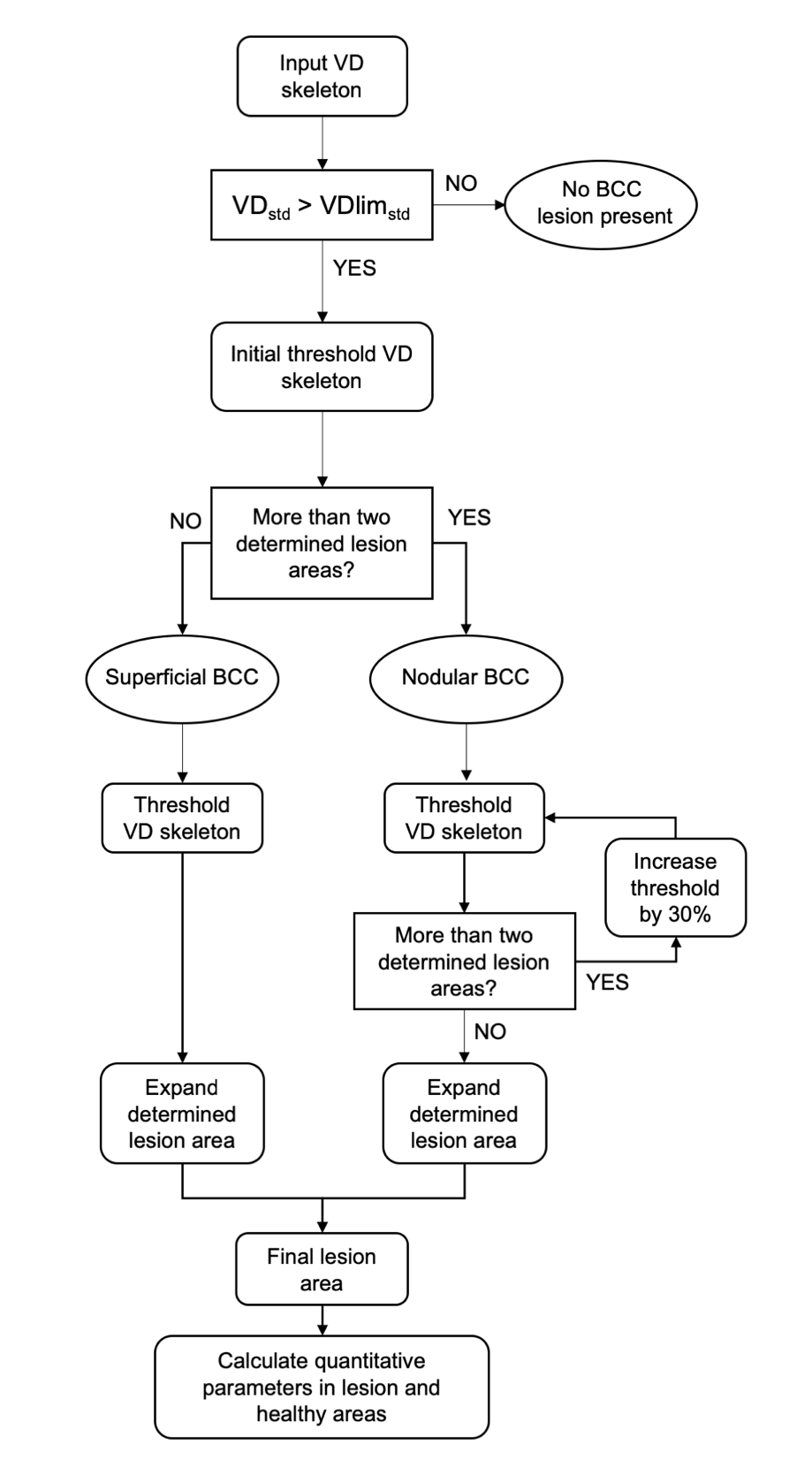

Supplement: Supplementary file 1 — Figure S1. General flowchart of the lesion area determination algorithm. ROI, region of interest; VD, vascular density. Figure S2. Flowchart highlighting specific steps in the algorithm, specifically how it is determined if a lesion is present; if present, how the lesion is determined to be superficial or nodular BCC; highlighting the difference in the algorithm when considering the two different BCC lesions. VD, vascular density; BCC, basal cell carcinoma; VDstd, SD of VD values within the skeleton; VDlimstd, threshold value for SD of VD values within the skeleton, equal to 4 × 10^−5^ in this preliminary study. [file JBIO-12-e201900131-s001.docx]
